# Stauntonia chinensis injection relieves neuropathic pain by increasing the expression of PSD‐95 and reducing the proliferation of phagocytic microglia

**DOI:** 10.1002/ibra.12140

**Published:** 2023-11-30

**Authors:** Wenwen Deng, Helin Zou, Li Qian, Senio Campos de Souza, Qian Chen, Song Cao

**Affiliations:** ^1^ Department of Cardiology Affiliated Hospital of Zunyi Medical University Zunyi Guizhou China; ^2^ Guizhou Key Lab of Anesthesia and Organ Protection Zunyi Medical University Zunyi Guizhou China; ^3^ Department of Pain Medicine Guizhou Provincial Orthopedics Hospital Guiyang Guizhou China; ^4^ Department of Chemistry University College London London UK

**Keywords:** microglia, neuroinflammation, neuropathic pain, PSD‐95, Stauntonia chinensis injection

## Abstract

Neuroinflammation induced by engulfment of synapses by phagocytic microglia plays a crucial role in neuropathic pain. Stauntonia chinensis is extracted from *Stauntonia chinensis* DC, which has been used as a traditional Chinese medicine to control trigeminal neuralgia or sciatica. However, the specific anti‐neuralgia mechanism of Stauntonia chinensis is unknown. In this study, the analgesic effect of Stauntonia chinensis injection (SCI) in mice with neuropathic pain and the possible mechanisms are explored. We find that a local injection of 0.1 mL Stauntonia chinensis for 14 days can considerably relieve mechanical hyperalgesia and thermal hyperalgesia in mice with sciatic chronic constriction injury (CCI). Immunofluorescence staining shows that SCI reduces neuroinflammation in the spinal cord of CCI mice. RNA sequencing reveals that the expression of postsynaptic density protein 95 (PSD‐95), a postsynaptic scaffold protein, is downregulated in the spinal cord of CCI mice, but upregulated after SCI administration. Immunofluorescence experiments also demonstrate that SCI administration reverses microglia proliferation and PSD‐95 downregulation in CCI mice. These data suggest that SCI relieves neuropathic pain by increasing the expression of PSD‐95 and reducing the proliferation of phagocytic microglia.

## Graphical abstract

Schematic diagram of the mechanism of Stauntonia chinensis injection (SCI) inhibiting neuropathic pain. Sciatic chronic constriction injury can cause inflammatory activation of spinal microglia, proliferation of phagocytic microglia, and prominent phagocytosis, resulting in a decrease in postsynaptic density protein 95 (PSD‐95). Simultaneously activated microglia release pro‐inflammatory cytokines, leading to an increase in neuroinflammation and an increase in neuronal action potentials (spikes), exacerbating neuropathic pain. SCI can inhibit the proliferation of phagocytic microglia and therefore increase PSD‐95 and inhibit the expression of inflammatory cytokines, as well as neuroinflammation and neuron spikes in the spinal cord.

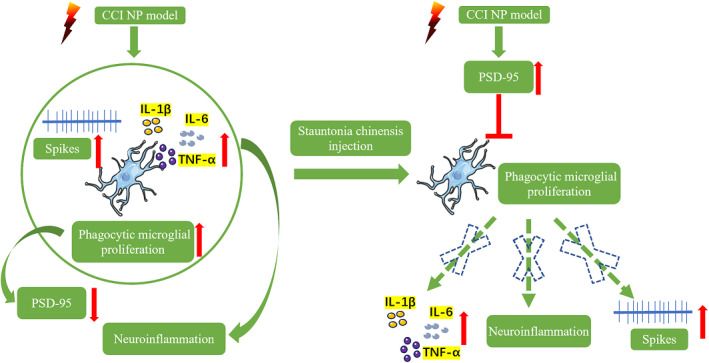

## 1 INTRODUCTION

Neuropathic pain is widely prevalent and poses challenges in terms of treatment.^1^ Better medications and technologies are needed to combat neuropathic pain. In experimental studies, a variety of animal models have been developed to mimic neuropathic pain. Among them, the sciatic chronic constriction injury (CCI) model can produce stable pain behavioral changes and is generally used in neuropathic pain studies.^2^ In addition, this nerve injury model induced changes of glial activation, synaptic plasticity, and neuroinflammation in the spinal cord.^3^ The development of neuropathic pain may be related to mechanisms that extend from the periphery to the central nervous system (CNS), involving the spinal cord, the brain, and descending pain regulating systems.^4^ Interneurons in the spinal dorsal horn (SDH) circuit receive synaptic signal input from different types of primary sensory neurons, and the SDH circuit plays a key role in regulating the transmission of sensory information.^5^, ^6^ Neuropathic pain is often accompanied by activation of spinal cord microglia and neuroinflammation and^3^ substantial changes in the SDH, and normal integration, processing, and transmission of sensory information are disrupted, which considerably contributes to the development of neuropathic pain.^7^ In addition, microglial activation increases neuronal synaptic phagocytosis, causing pathological symptoms such as depression, Alzheimer's disease (AD),^8^ and so forth, contributing to the development and maintenance of neuropathic pain.^9^

As one of the treatments for neuropathic pain, traditional Chinese medicine is considered as an alternative choice.^10^ Stauntonia chinensis injection (SCI) is produced from *Stauntonia chinensis* DC, a plant of the wood family that is an evergreen herb, mostly grown in southern China, with the Chinese name “Ye Mu Gua.” Studies have reported that SCI has a good clinical analgesic effect^11^ and has been used as an anti‐neuralgia medicine in patients with trigeminal neuralgia and sciatica.^12^ Some studies have shown that SCI also has anti‐nociceptive and anti‐inflammatory activities, suggesting that SCI alleviates pain by inhibiting the inflammatory response.^13^, ^14^ The rich triterpene glycosides in Stauntonia Chinensis may be converted into their respective aglycones, thus inhibiting the release of inflammatory mediators in the body. This may explain the clinical value of the anti‐inflammatory effect of SCI.

Postsynaptic density protein 95 (PSD‐95) plays a key role in neuropathic pain by interacting with other cytokines in SDH.^15^ Studies have shown that in the spinal cord of AD mice, the expression of PSD‐95 is reduced, and the activation of microglial cells increases neuroinflammation.^16^ In the hippocampal dentate gyrus of chronic social defeat stress mice, the density of phagocytic microglial cells increased and the activated microglial cells contained more PSD‐95 phagocytic spots.^17^ The activation of microglial cells causes neuroinflammation, which is closely related to the phagocytosis of neuronal synapses, and PSD‐95 is involved in the process of synaptic phagocytosis.

In this study, by SCI administration at the site of sciatic nerve injury in CCI mice, we demonstrated that SCI alleviated both mechanical and thermal hyperalgesia in mice. We also found that SCI inhibited the activation of microglial cells in the spinal cord of CCI mice, alleviated neuroinflammation, and modulated the synaptic phagocytosis of microglial cells by regulating the expression levels of PSD‐95 in SDH.

## 2 MATERIALS AND METHODS

### 2.1 Animals

This study was approved by the Laboratory Animal Welfare & Ethics Committee of Zunyi Medical University (ZMU21‐2210‐002). Animal studies were performed following the Guide for the Care and Use of Laboratory Animals. Male C57/BL6 mice (8–12 weeks old, 25–30 g) were purchased from the Tianqin Biotechnology (Changsha, China). Three to four mice were accommodated in a cage at a constant room temperature of 23 ± 2°C and relative humidity of 55% ± 2% with a 12:12 h light/dark cycle. Food and water were freely accessed. All mice were allowed to adapt to the environment for 1 week before the experiments. Mice were randomly divided into four groups: sham group, CCI group, CCI + normal saline (NS) group, and CCI + SCI group.

### 2.2 CCI model

**Figure 1:**
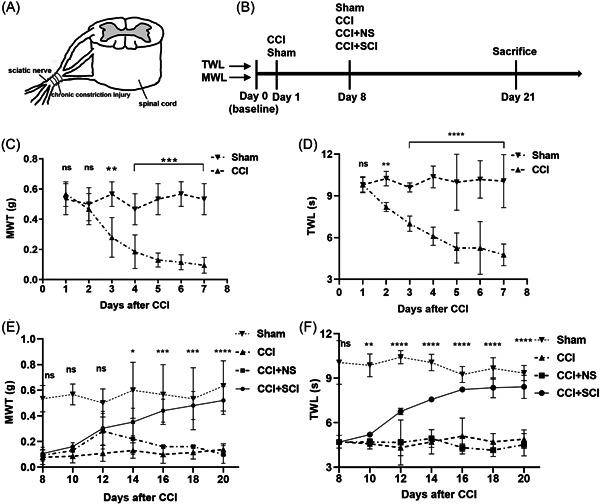
Local administration of SCI adjacent to injured sciatic nerve alleviates neuropathic pain in CCI mice. (A) Ligation of one side of the sciatic nerve. (B) Experimental protocol. (C) Changes of MWT in mice after CCI. **p < 0.01; ***p < 0.001. (D) Changes of TWL in mice after CCI. **p < 0.01, ****p < 0.0001. (E) The MWT increased in CCI mice after SCI treatment. ns, not significant. CCI + SCI group versus CCI + NS group (*p < 0.05; ***p < 0.001; ****p < 0.0001). (F) The TWL was prolonged in CCI mice after SCI treatment. ns, not significant. CCI + SCI group versus CCI + NS group (**p < 0.01, ****p < 0.0001). *n* = 8, mean ± SD, one‐way ANOVA, one‐way repeated ANOVA. CCI, chronic constriction injury; MWT, mechanical withdrawal thresholds; NS, normal saline; SCI, Stauntonia chinensis injection; TWL, thermal withdrawal latency.

Mice were anesthetized with 2%–3% (vol) isoflurane and placed on a warm mat. The surgical site was carefully prepared with scissors to reduce contamination and avoid damaging the skin of the mice. A 1 cm longitudinal incision was made on the left leg at the proximal end of the knee joint with a scalpel, the biceps femoris muscle was separated to expose the sciatic nerve, and the sciatic nerve was ligated with four ligatures with 6‐0 chromic gut sutures; a strength that just causes the ipsilateral hind limb to twitch is appropriate, with each ligature approximately 1 mm apart (Figure 1A). Then, the incisions were sutured layer by layer. In the sham group, only the sciatic nerve and its branches were exposed without any ligation.

### 2.3 Drug administration

Seven days after CCI insult, mice were randomly divided into four groups: sham group, CCI group, CCI + NS group, and CCI + SCI group. Animals in the CCI + SCI group were locally injected with 0.1 mL of Stauntonia Chinensis (Xinfeng Pharmaceutical Co., Ltd.) at the ligated sciatic nerve at 10 a.m. and 10 p.m. every day from Day 8 to Day 21, while the CCI + NS mice were injected with the same amount of NS in the same position around the sciatic nerve at the same time each day. SCI or NS was administered continuously for 14 days (Figure 1B). Animals in the sham and CCI groups were not administered any injection.

### 2.4 Von Frey's test for mechanical allodynia and Hargrave's test for thermal hyperalgesia

For the assessment of mechanical allodynia, we used Von Frey filaments with ascending forces expressed in grams.^18^ Mice were placed on a mesh suspension plate and allowed to adapt to the environment for 30 min (min). Mechanical withdraw thresholds (MWTs) were tested using a Von Frey filament kit (Danmic Global) with the up‐down method.^19^

For the assessment of thermal hyperalgesia, animals were placed in an individual plexiglass enclosure on a transparent glass surface maintained at 25°C and allowed to adjust to the environment for 30 min. The thermal withdrawal latency (TWL) was detected using a plantar radiant heat pain tester (IITC), and the device was activated by placing the beam vertically under the left hind paw. Mice were recorded when they raised their hind paws for exactly the time of heat withdrawal latency. The cut‐off time to prevent left foot injury was set at 20 s. The average of five tests, five times for each mouse with a 5 min interval, was considered to be the thermal exit latency. Left paws were tested at 5 min intervals for a total of five trials.

### 2.5 In vivo extracellular recordings of wide‐dynamic‐range neurons in the SDH

Mice were anesthetized with isoflurane and fixed in a stereotaxic frame (David Kopf Instruments). The level of anesthesia was maintained by administering a constant amount of isoflurane (1.5% in oxygen) using a precision vaporizer throughout the experiment. The depth of anesthesia was monitored closely by testing the corneal blink, hindpaw withdrawal, and tail‐pinch reflexes, and these had to have disappeared to ensure a constant level of anesthesia.

Extracellular recordings of individual neurons were performed in the deep dorsal horn (300–1000 μm) of the lumbar enlargement of the spinal cord (L5/6) using a single‐channel electrode (Monopolar Tengsten Electrode; WE3003X, Microprobes for Life Science) connected to a multi‐channel fiber photometry system (Thinkertech Nanjing Bioscience Inc.). The recorded signals were amplified, displayed on an analog storage oscilloscope, fed into a window discriminator, digitized by an interface (CED 1401+; Cambridge Electronics Design), and recorded on a computer (Pentium PC). The neurons were characterized by their responses to the following stimuli applied to the most responsive sites of the receptive field in the hind paw: innocuous BRUSH (brushing the skin with a soft‐hair artist's brush in a stereotyped manner; 1 stroke); innocuous PRESS (100 g/6 mm^2^), which is not painful when applied to the skin in humans); and noxious PINCH (300 g/6 mm^2^), which is painful without causing overt damage to the skin. Spike size and configuration were continuously monitored on the oscilloscope using Spike‐2 software (CED) to confirm that the same neuron was recorded and that the relationship of the recording electrode to the neuron remained constant. All cells included in this study were wide‐dynamic‐range (WDR) neurons, which responded consistently to innocuous stimuli but more strongly to noxious stimuli.

The recorded activity (spikes per s) was analyzed offline from peristimulus rate histograms using Spike2 software (CED). Background activity was subtracted from the total activity during stimulation to calculate “net” evoked responses.

### 2.6 Enzyme‐linked immunosorbent assay (ELISA)

Mice were anesthetized with 3% (vol) isoflurane and perfused with phosphate‐buffered saline (PBS), and the lumbar enlargement of the spinal cord was collected and stored at −80°C. Before the ELISA assay, spinal cord tissue was homogenized with 0.1 M PBS and protease inhibitor cocktail (20 μL/100 mg tissue, P8340; Sigma) and centrifuged at 5000*g* for 10 min at 4°C. Then, the supernatant was collected and the total protein content was determined using the bicinchoninic acid (BCA) assay kit (23225; Thermo Fisher Scientific). The concentration of the supernatant was normalized to the protein content, and the levels of tumor necrosis factor α (TNF‐α), interleukin‐6 (IL‐6), and interleukin‐1β (IL‐1β) were detected using the ELISA kit (Beyotime) according to the manufacturer's instructions. The optical density at 450 nm of each well was measured. The concentration of the target protein in the samples was determined by comparing the absorbance values to the standard curve generated from known concentrations of the target protein.

### 2.7 Immunofluorescence

Mice were anesthetized with 3% isoflurane and perfused with PBS and 4% paraformaldehyde. The spinal cord and the sciatic nerve on the operative side were harvested immediately, and the tissue was then transferred to 30% sucrose for dehydration. After the tissue was submerged to the bottom, the tissue was embedded with OCT compound and then cut into 30 μm thick sections using a freezing microtome (Leica). They were subsequently washed three times with PBS containing 0.3% Triton X‐100 (5 min), and then blocked with 1% bovine serum albumin and 0.3% Triton X‐100 in PBS for 2 h. Afterward, the sections were incubated with rabbit anti‐IBA‐1 (ionized calcium binding adapter molecule 1) (1:1000; Kaz), rabbit anti‐PSD‐95 (1:100; Santa Cruz Biotechnology), mouse anti‐TNF‐α (1:1000; Abcam), and mouse anti‐IL‐1β (1:1000; Santa Cruz Biotechnology) antibody and refrigerated overnight at 4°C. After removing the primary antibodies with PBS (5 min each, three times), secondary antibodies were added to the sections, goat anti‐rabbit IgG H&L (DyLight 488) (1:1000; Abcam) and goat anti‐mouse IgG H&L (Alexa Fluor 594) (1:1000; Abcam), and incubated for 2 h at room temperature without light. Finally, sections were counterstained with DAPI and then photographed under a fluorescence microscope (Olympus). To quantify the expression of IBA‐1, PSD‐95, IL‐1β, and TNF‐α, Image J software (v.1.52A; NIH) was used to delineate the spinal cord images with size‐standardized regions of interest (ROIs),^20^ and the percentage of fluorescent‐positive regions was quantified with Image J (v.1.52A; NIH). In particular, the threshold was set and standardized across images to maximize the true protein expression signal for quantification. The percentage of positive area was calculated: positive area% = (the total pixel number of the target protein/the pixel number with the total unfiltered pixel number in the ROI) × 100.

For BV2 microglial immunofluorescence, the cells were first fixed with 4% paraformaldehyde for 10 min, followed by three washes with 0.01 M PBS. Then, cells were incubated with 10% donkey serum‐containing blocking solution for 30 min at room temperature, followed by incubation of the primary antibody, rabbit anti‐IBA‐1, solution containing 10% donkey serum, 0.3% triton X‐100, in 0.01 M PBS at 4°C for 24 h. After three washes with PBS, the cells were then incubated with Alexa Fluor‐488‐conjugated donkey rabbit IgG (1:500; Molecular Probes) for 12 h at room temperature. With DAPI nuclear counterstaining for 10 min, they were photographed under a confocal microscope. Image J (v.1.52A; NIH) was used to calculate the proportion of cell fluorescence. The mean intensity of IBA‐1 in each group is the sum of all fluorescence values in the field of view (set as 1 in the control group). IBA‐1 intensity density in each group is the sum of all fluorescence values/IBA‐1‐positive cell number in the field of vision.

### 2.8 Cell culture and drug treatments

The murine BV2 microglial cell line (Lot: 5HQ0GICW2X; Procell) was provided by Professor Zhaoqiong Zhu at Zunyi Medical University. The cells were cultured in DMEM high‐glucose complete medium (Cat: 10‐013‐CVRC; Corning), supplemented with 10% fetal bovine serum (Cat: 04‐0011‐1ACS; Biological Industries) and a 1% penicillin‐streptomycin solution at 37°C in a humidified incubator with 5% CO_2_ in T25 flasks. Once the cells reached over 80% confluence, they were plated in 12‐well plates at a density of 5 × 10^3^ cells per well, which was conducted 24 h after the cells were seeded. BV2 microglial cells were induced to an inflammatory state in vitro by stimulating them with 0.5 μg/mL lipopolysaccharide (LPS).^21^ To investigate the effect of SCI on LPS‐induced neuroinflammation in BV2 microglial cells, the cells were divided into four groups: control group, LPS group, LPS + PBS group, and LPS + SCI group. In the LPS + SCI and LPS + PBS groups, SCI (0.1 mL/mL)^22^ and PBS (0.1 mL/mL) were added along with LPS to the cells, respectively. After 24 h of treatment, microglial biomarker IBA‐1 was detected using immunofluorescence staining.^20^ IBA‐1 is a calcium‐binding protein that is specific to microglia/macrophages and is commonly used to evaluate microglia cell activation.^23^

### 2.9 Library preparation for transcriptome sequencing

Mouse spinal cord was isolated, and total RNA was extracted using RNAiso Plus (TaKaRa). RNA degradation and contamination were monitored on 1% agarose gels. RNA purity was checked using the NanoPhotometer® spectrophotometer (IMPLEN). RNA integrity was assessed and RNA was quantified using the RNA Nano 6000 Assay Kit of the Bioanalyzer 2100 system (Agilent Technologies).

A total amount of 1 µg RNA per sample was used as input material for the RNA sample preparations. Sequencing libraries were generated using the NEBNext® UltraTM RNA Library Prep Kit for Illumina (NEB) following the manufacturer's recommendations and index codes were added to attribute sequences to each sample. Briefly, mRNA was purified from total RNA using poly‐T oligo‐attached magnetic beads. Fragmentation was carried out using divalent cations at high temperatures in NEBNext First Strand Synthesis Reaction Buffer (5X). First‐strand complementary DNA (cDNA) was synthesized using a random hexamer primer and M‐MuLV Reverse Transcriptase (RNase H^−^). Second‐strand cDNA synthesis was subsequently performed using DNA Polymerase I and RNase H. The remaining overhangs were converted into blunt ends via exonuclease/polymerase activities. After adenylation of 3′ ends of DNA fragments, NEBNext Adaptor with a hairpin loop structure was ligated to prepare for hybridization. In order to preferentially select cDNA fragments 250–300 bp in length, the library fragments were purified using the AMPure XP system (Beckman Coulter). Then, 3 µL of USER Enzyme (NEB) was used with size‐selected, adaptor‐ligated cDNA at 37°C for 15 min, followed by 5 min at 95°C before polymerase chain reaction (PCR). Then, PCR was performed with Phusion High‐Fidelity DNA polymerase, Universal PCR primers, and the Index (X) Primer. Finally, PCR products were purified (AMPure XP system) and library quality was assessed on the Agilent Bioanalyzer 2100 system.

### 2.10 Clustering and sequencing

Clustering of the index‐coded samples was performed on a cBot Cluster Generation System using the TruSeq PE Cluster Kit v3‐cBot‐HS (Illumina) according to the manufacturer's instructions. After cluster generation, the library preparations were sequenced on an Illumina Novaseq platform and 150 bp paired‐end reads were generated.

### 2.11 Quantification of gene expression levels

FeatureCounts v1.5.0‐p3 was used to count the reads numbers mapped to each gene. Then, FPKM of each gene was calculated based on the length of the gene and reads count mapped to this gene. FPKM, the expected number of Fragments Per Kilobase of transcript sequence per millions base pairs sequenced, considers the effect of sequencing depth and gene length for the reads count at the same time, and is currently the most commonly used method for estimating gene expression levels.

### 2.12 Differential expression analysis

Differential expression analysis of two conditions/groups (two biological replicates per condition) was performed using the DESeq. 2R package (1.16.1). DESeq. 2 provides statistical routines for determining differential expression in digital gene expression data using a model based on the negative binomial distribution. The resulting *p* values were adjusted using Benjamin and Hochberg's approach to control the false discovery rate. Genes with an adjusted *p* value <0.05 found by DESeq. 2 were assigned as differentially expressed genes (DEGs).

### 2.13 Gene Ontology (GO) and Reactome enrichment analysis of DEGs

GO enrichment analysis of DEGs was carried out using the clusterProfiler (v4.0) package in R software, in which gene length bias was corrected. GO terms with corrected *p* values less than 0.05 were considered significantly enriched by DEGs.

Reactome is a database resource for understanding high‐level functions and utilities of the biological system, such as the cell, the organism, and the ecosystem, from molecular‐level information, especially large‐scale molecular data sets generated by genome sequencing and other high‐throughput experimental technologies. We used clusterProfiler package to test the statistical enrichment of DEGs in Reactome pathways.

### 2.14 Western blot

The L3‐6 (lumbar enlargement) spinal cords of mice were collected, lysed in radioimmunoprecipitation assay buffer, and centrifuged at 12,000*g* for 20 min at 4°C. The BCA protein assay kit was used to determine the total protein concentration. Lysates were mixed with 5× sodium dodecyl sulfate‐polyacrylamide gel electrophoresis (SDS‐PAGE) loading buffer and boiled at 95°C for 10 min. Different groups of samples containing approximately 20 mg of protein were separated by 12% SDS‐PAGE and then the proteins in the gels were transferred to polyvinylidene fluoride (PVDF) membranes. Afterward, the PVDF membrane was blocked on a shaker for 2 h at room temperature with the addition of 5% (w/v) nonfat milk in PBS with Tween 20 (PBST) buffer. Subsequently, primary antibodies including rabbit anti‐PSD‐95 (1:1000; Santa Cruz Biotechnology) and rabbit anti‐β‐actin (1:5000; Proteintech) were added and incubated overnight at 4°C. Then, the membrane was washed three times for 10 min each time and incubated with sheep anti‐rabbit horseradish peroxidase‐conjugated secondary antibody (1:2000; Proteintech) for 2 h. Finally, after washing as described previously, antigen–antibody complexes were detected with enhanced chemiluminescence substrates. Images were collected and then used for the final protein expression assays using Image Lab™ (v4.1) software and normalized to loading controls.

Protein expression levels were calculated as relative expression = optical density value of target protein/to optical density value of β‐actin.

### 2.15 Statistical analysis

GraphPad Prism 9.0 (GraphPad Software) and SPSS 19.0 (IBM) were used for statistical analysis. Data were expressed as mean ± standard deviation (SD). Statistically, differences between two groups at the same time points were analyzed using unpaired *t*‐tests. Differences among multiple groups at the same time point were analyzed by one‐way analysis of variance (ANOVA), followed by Tukey's multiple comparisons tests. One‐way repeated ANOVA was used to analyze the differences of multiple time points in the same group, followed by paired *t*‐tests to compare differences between two time points. Normal distributions were tested using the *F*‐test. The Kruskal–Wallis check was used for multiple cluster comparisons that failed to change to traditional homogeneity and homogeneity of variance. Immunofluorescence images were analyzed statistically using Image J (NIH, Washington, USA). *p* < 0.05 was considered statistically significant.

## 3 RESULTS

### 3.1 SCI treatment adjacent to the injured sciatic nerve alleviated thermal hyperalgesia and mechanical allodynia

Pain thresholds were measured daily in sham‐operated mice and CCI mice, and the CCI mice showed thermal hyperalgesia and mechanical allodynia compared with the sham‐operated mice from day 2 (Figure 1C,D, *p* < 0.01). Compared with the CCI group and the CCI + NS group, the MWT and TWL of the CCI + SCI group were significantly increased from the sixth and second day of SCI administration, respectively, and continuous treatment of SCI led to sustained relief of thermal hyperalgesia and mechanical allodynia (Figure 1E,F, *p* < 0.05).

### 3.2 SCI inhibited firing of WDR neurons in the SDH of CCI mice

**Figure 2:**
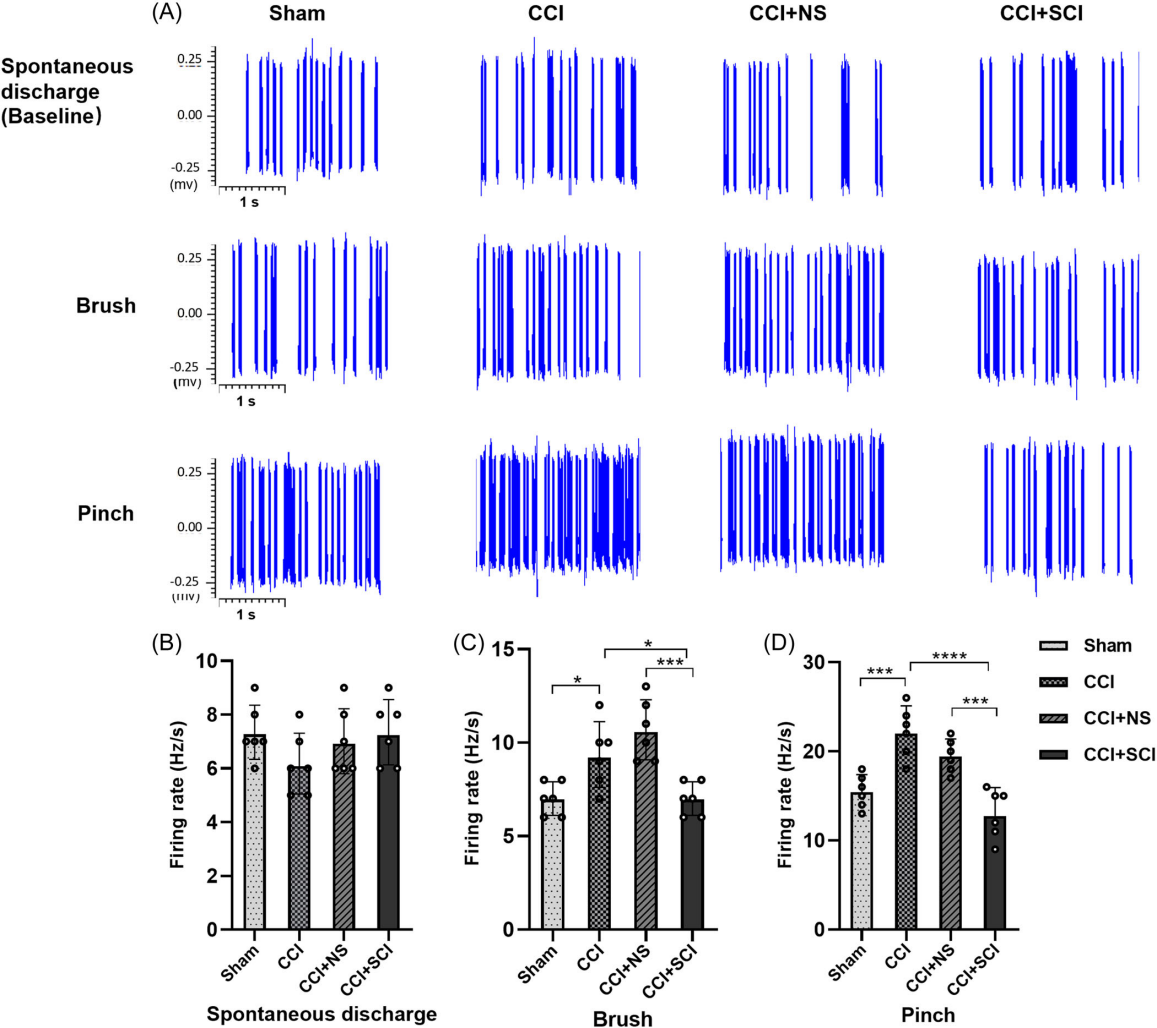
Firing frequency of WDR neurons in SDH. (A) Firing frequency of spinal cord WDR neurons in each group of mice under different stimuli. (B–D) Changes of the firing frequency of WDR neurons in SDH under different stimuli among the sham, CCI, CCI + NS, and CCI + SCI groups. *n* = 6, mean ± SD, one‐way ANOVA. CCI, chronic constriction injury; NS, normal saline; SCI, Stauntonia chinensis injection; WDR, wide dynamic range. *p < 0.05; ***p < 0.001; ****p < 0.0001. [Color figure can be viewed at wileyonlinelibrary.com]

We performed extracellular single‐unit recordings of SDH WDR neurons. It was observed that there was no significant difference in the spontaneous discharge frequency of WDR neurons in the spinal cord among the four groups (Figure 2A,B, *p* > 0.05). Upon stimulation of the sole of the operated side of the mouse with a brush, the WDR discharge frequency of spinal cord neurons in the CCI group was increased significantly compared with the sham group (*p* < 0.05), while the WDR discharge frequency of spinal cord neurons in the CCI + SCI group showed marked reduction compared with the CCI and CCI+NS groups (Figure 2A,C, *p* < 0.05, *p* < 0.001). Similarly, upon application of noxious stimulation to the soles of the mice, the WDR discharge frequency of spinal cord neurons in the CCI group was significantly increased compared with the sham group (*p* < 0.001), whereas the frequency after SCI treatment showed a prominent decrease relative to that in the CCI and CCI+NS groups (Figure 2A,D, *p* < 0.0001, *p <* 0.001).

### 3.3 SCI inhibited CCI‐induced phagocytic microglia in the SDH and reduced spinal cord neuroinflammation

**Figure 3:**
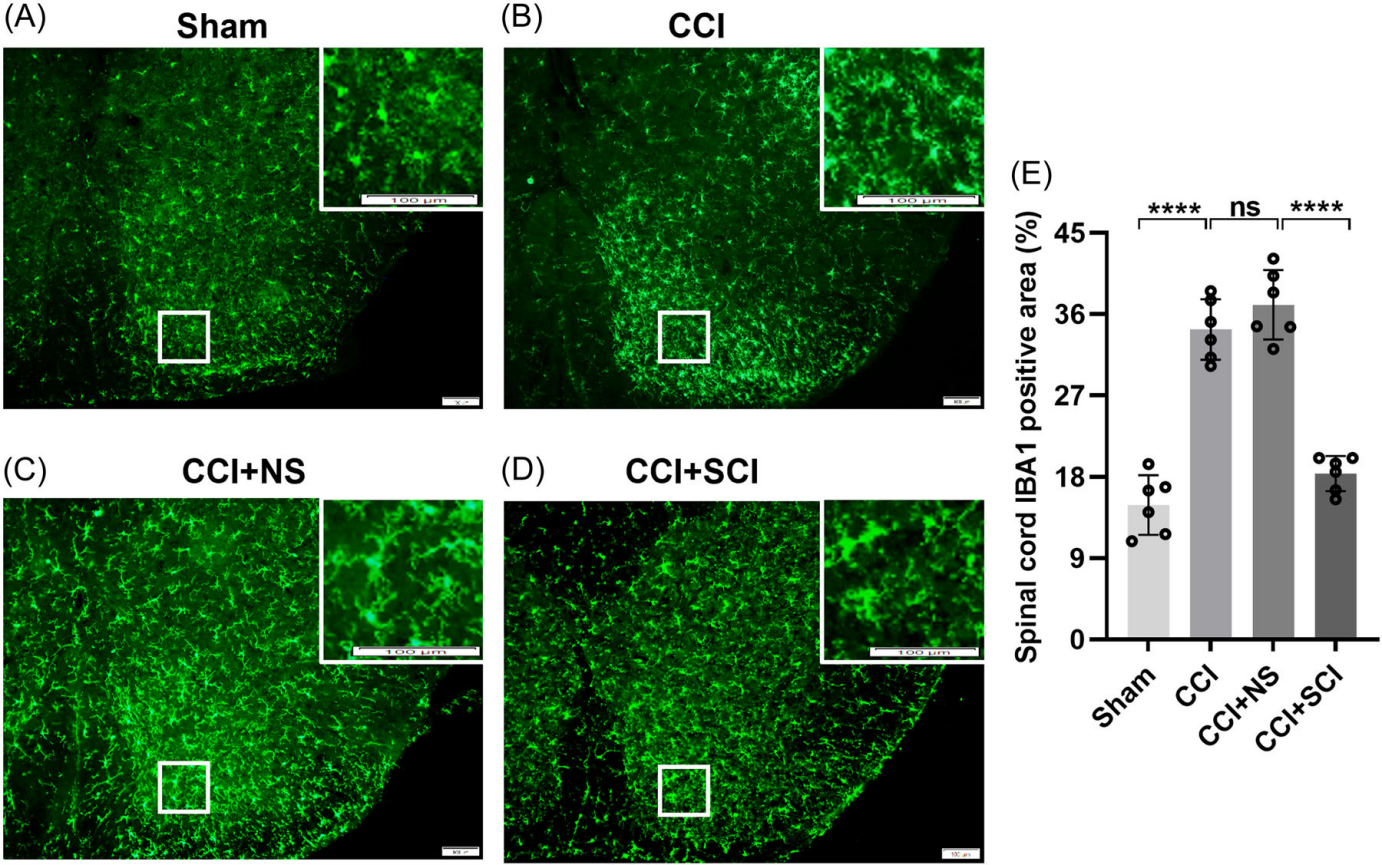
Phagocytic microglial in the SDH. (A–D) Immunofluorescence images of IBA‐1 in the SDH of mice in the sham, CCI, CCI + NS, and CCI + SCI groups. Scale bars = 100 μm. Green, IBA‐1‐positive cells. White rectangles indicate the areas of interest. (E) Proportions of IBA‐1 expression in the SDH among the sham, CCI, CCI + NS, and CCI + SCI groups. *n* = 6, mean ± SD, one‐way ANOVA. CCI, chronic constriction injury; IBA‐1, ionized calcium binding adapter molecule 1; ns, not significant; NS, normal saline; SCI, Stauntonia chinensis injection. ****p < 0.0001. [Color figure can be viewed at wileyonlinelibrary.com]

Previous research has reported abnormal activation of microglia in the phagocytosis of neuronal synapses.^9^ We used immunofluorescence to evaluate morphological changes in microglia‐mediated synaptic phagocytosis induced by CCI. We observed that compared with the sham group, widespread proliferation of microglia in the SDH was observed in the CCI group, with increased density and altered morphology characterized by thickened processes, enlarged cell bodies, and retracted protrusions (Figure 3A,B,E, *p* < 0.0001). However, there was significantly suppressed proliferation of microglia in the SDH of mice in the CCI + SCI group (Figure 3D), indicated by the decreased expression of IBA‐1 in the SDH of mice in the CCI+SCI group relative to that of the CCI+NS group (Figure 3C–E, *p* < 0.0001). These data suggested that SCI suppressed the proliferation of synaptic phagocytic microglia.

### 3.4 SCI inhibits LPS‑induced microglial cell proliferation in vitro

**Figure 4:**
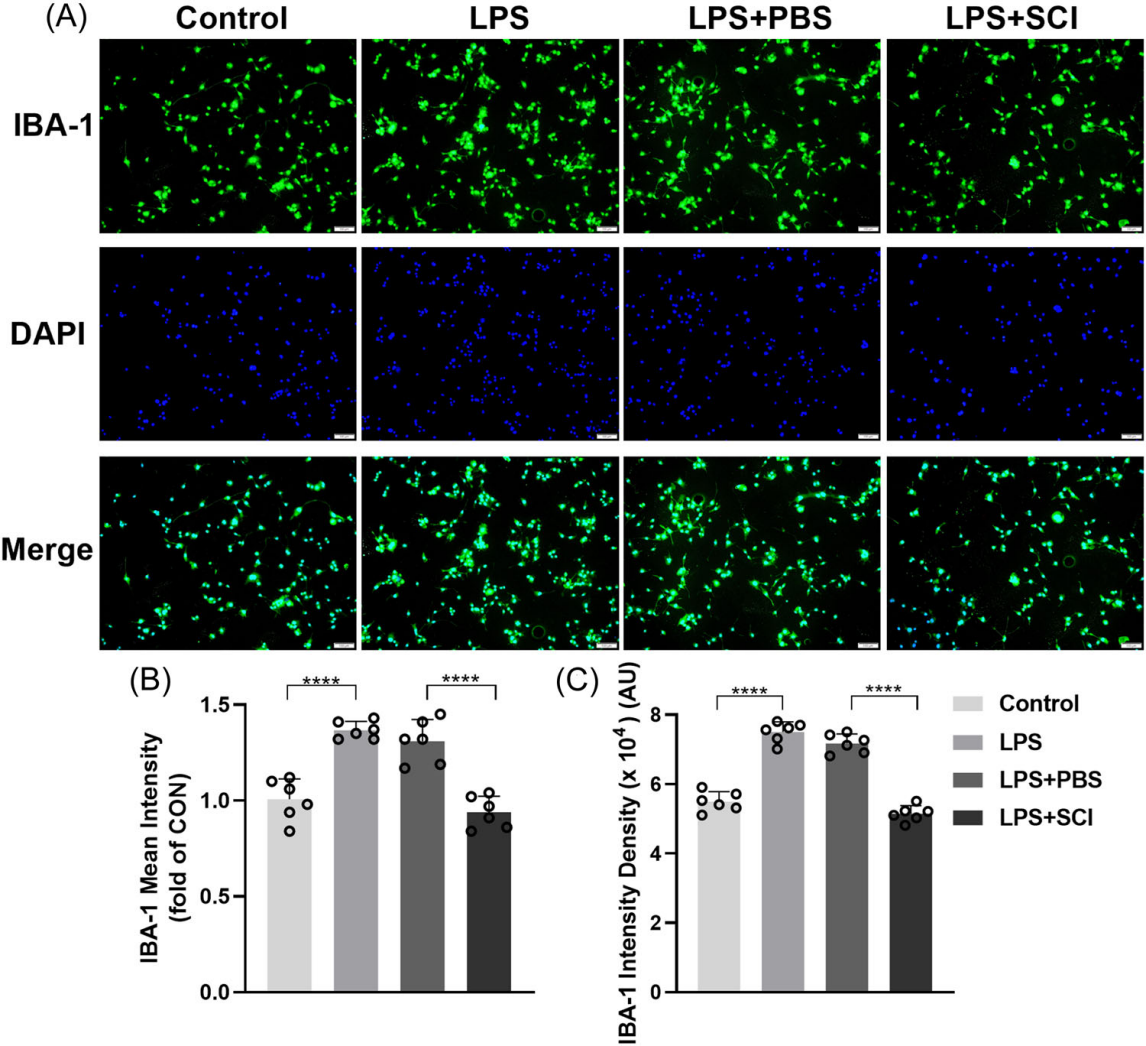
SCI inhibits the in vitro proliferation of LPS‐induced BV2 microglia cells. (A) Immunofluorescence images show BV2 microglial cells labeled with the anti‐IBA‐1 antibody after treatment with LPS and SCI. Green, IBA‐1‐positive cells. Blue, DAPI. Scale bars = 100 μm. (B) Mean intensity fold change of IBA‐1 expression in the LPS, LPS + PBS, and LPS + SCI groups relative to the control group. (C) Intensity density of IBA‐1‐positive cells in the control, LPS, LPS + PBS, and LPS + SCI groups relative to the control group. *n* = 6, mean ± SD, one‐way ANOVA. IBA‐1, ionized calcium binding adapter molecule 1; LPS, lipopolysaccharide; PBS, phosphate‐buffered saline; SCI, Stauntonia chinensis injection. ****p < 0.0001. [Color figure can be viewed at wileyonlinelibrary.com]

A study has shown that 0.5 μg/mL LPS promotes the proliferation of BV2 microglia cells.^21^ We evaluated the effect of SCI on LPS‐induced microglial cell proliferation by culturing BV2 microglia cells in LPS (0.5 μg/mL) and SCI (0.1 mL/mL) for 24 h. The results of cell immunofluorescence indicated that BV2 microglial cells showed significant activation in response to LPS stimulation compared to the control group, with an increase in cell volume and rounding, but SCI treatment (0.1 mL/mL) notably inhibited LPS‐induced BV2 microglia cell proliferation in comparison with LPS + PBS microglial cells (Figure 4A–C, *p* < 0.0001).

### 3.5 Phagocytic microglia contribute to neuroinflammation in the spinal cord

**Figure 5:**
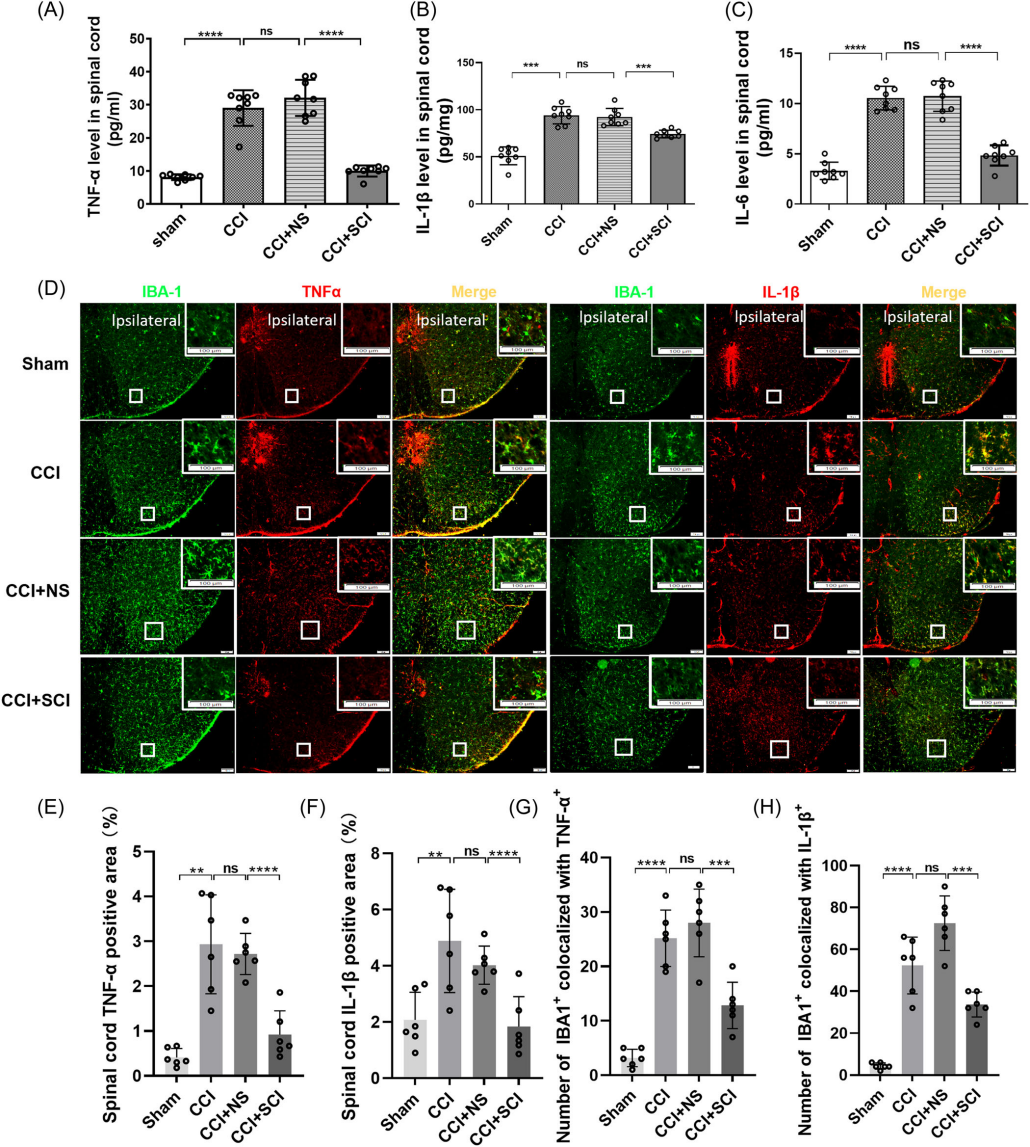
Expression of inflammatory cytokines in the spinal cord and colocalization of TNF‐α and IL‐1β with microglia marker IBA‐1. (A–C) Levels of TNF‐α, IL‐6, and IL‐1β in the spinal cord of mice in the sham, CCI, CCI + NS, and CCI + SCI groups. *n* = 8, mean ± SD, one‐way ANOVA. (D) Colocalization of TNF‐α (red) or IL‐1β (red) with IBA‐1 (green) in the SDH. White rectangles indicate the areas of interest and enlarged images. Scale bars = 100 μm. (E, F) TNF‐α and IL‐1β expression was detected in the SDH of the sham, CCI, CCI + NS, and CCI + SCI groups. (G, H) Quantification histograms showed colocalization of IBA‐1 with TNF‐α, or IL‐1β in the four groups. Scale bars = 100 μm, *n* = 6, mean ± SD, one‐way ANOVA. CCI, chronic constriction injury; IBA‐1, ionized calcium binding adapter molecule 1; IL‐1β, interleukin‐1β; IL‐6, interleukin‐6; ns, not significant; NS, normal saline; SCI, Stauntonia chinensis injection; TNF‐α, tumor necrosis factor α. **p < 0.01; ***p < 0.001; ****p < 0.0001. [Color figure can be viewed at wileyonlinelibrary.com]

Chronic neuropathic pain is closely related to neuroinflammation. We also used ELISA to determine the content of inflammatory factors in the spinal cord. The results showed that compared with the sham group, the contents of IL‐1β, IL‐6, and TNF‐α in the spinal cord of the CCI group were significantly increased (Figure 5A–C, *p* < 0.001, *p* < 0.0001). The contents of IL‐1β, IL‐6, and TNF‐α in the SDH of the CCI+SCI group were significantly decreased compared with those in the CCI + NS group (Figure 5A–C, *p* < 0.001, *p* < 0.0001). Phagocytic microglial cell number and activity are closely related to neuroinflammation.^20^, ^24^ The results of immunofluorescence showed that the proportion of the fluorescence area of inflammatory cytokines in the SDH of mice in the CCI group was significantly increased compared with that of the sham group. After local administration of SCI or NS to CCI mice, the proportion of the fluorescence area of inflammatory factors in the SDH of mice in the CCI + SCI group was decreased compared with that in the CCI+NS group (Figure 5D–F, *p* < 0.0001). Moreover, our results also showed that the two inflammatory cytokines, TNF‐α and IL‐1β, were mostly colocalized with microglia marker IBA‐1, both of which were positively correlated with the cell number and activation of microglia (Figure 5D,G,H, *p* < 0.001, *p* < 0.0001), indicating that increased phagocytic microglia caused neuroinflammation in the spinal cord.

### 3.6 The expression of PSD‐95 was upregulated in the spinal cord of SCI‐treated CCI mice

**Figure 6:**
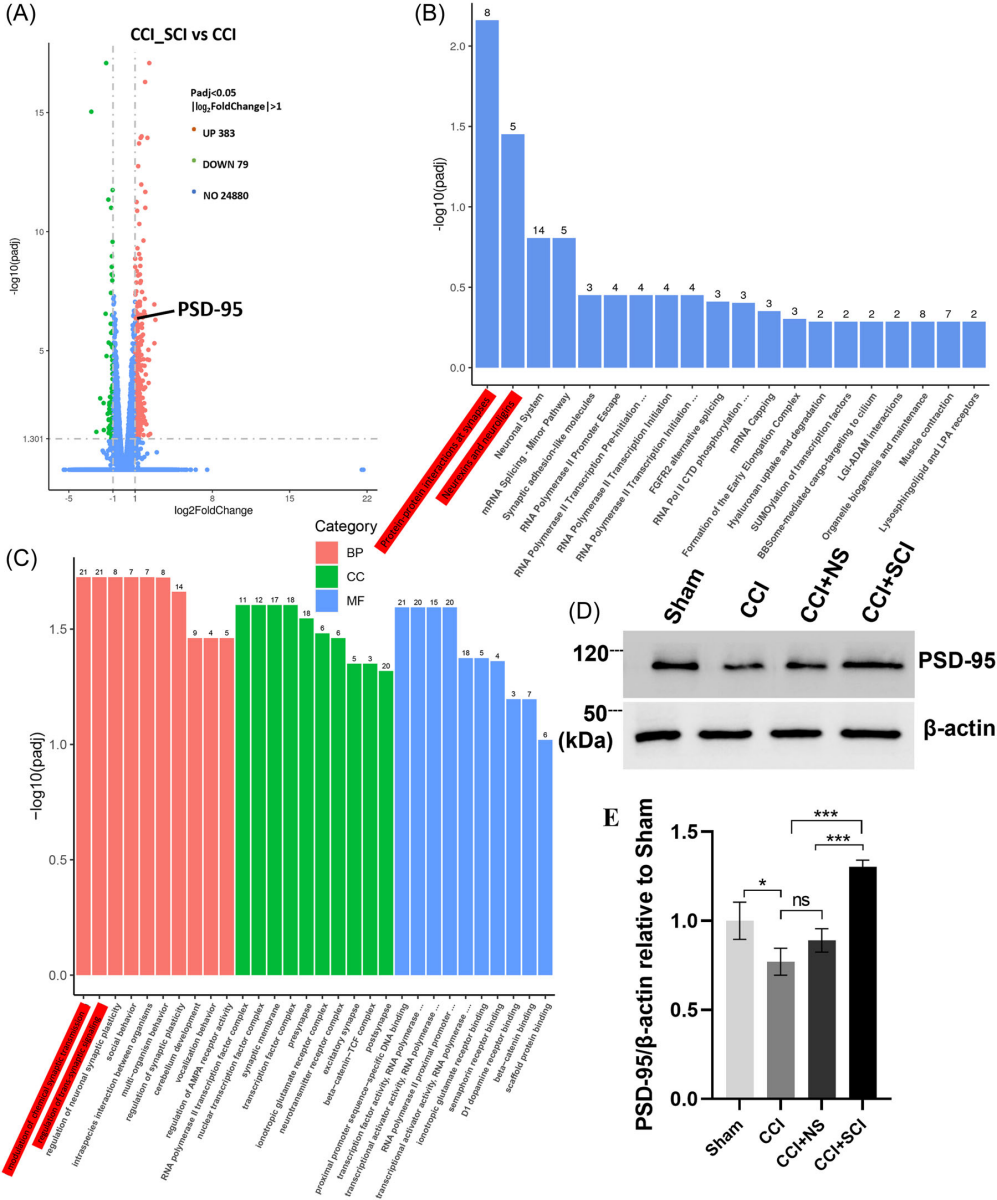
Biological function analysis of DEGs screened by RNA sequencing. (A) Volcano plot showing the up‐ or downregulated DEGs. (B) Reactome pathway enrichment analysis. The most significant 20 Reactome pathways are shown. (C) GO annotation enrichment analysis. The top 10 high enrichment score terms in biological process, cellular components, and molecular functions. (D) Expression of PSD‐95 in the spinal cord detected by Western blot. (E) Western blot quantification results showing the protein levels of PSD‐95 in each group. *n* = 3, mean ± SD, one‐way ANOVA. BP, biological process; CC, cellular components; CCI, chronic constriction injury; GO, Gene Ontology; MF, molecular functions; NS, normal saline; PSD‐95, postsynaptic density protein 95; SCI, Stauntonia chinensis injection. *p < 0.05; ***p < 0.001. [Color figure can be viewed at wileyonlinelibrary.com]

Gene enrichment analysis is a method to analyze gene expression information. Enrichment is performed to classify genes by Component, Function, Pathway, and other properties, and to classify and annotate gene sets composed of genes with the same or similar functions. The RNA sequencing results showed that 383 genes were upregulated (PSD‐95 included) and 79 genes were downregulated in the spinal cord of CCI+SCI mice, compared with the CCI mice (Figure 6A). The Reactome database is a collection of responses and biological pathways in human and other model species. The enrichment results of the Reactome pathway showed that the DEGs were mainly involved in “protein–protein interactions at synapses” and “neurexins and neuroligins” (Figure 6B). Meanwhile, GO functional enrichment showed that the DEGs were primarily enriched in BP terms such as “regulation of transynaptic signaling,” “neuronal synaptic plasticity,” “synaptic plasticity,” “AMPA receptor activity,” and “neurotransmitter receptor activity” (Figure 6C). We noticed that PSD‐95 highly coincided in GO functional enrichment significant items and the Reactome enrichment significant pathways. Meanwhile, Western blot results also showed that the expression of PSD‐95 increased in the spinal cord of mice in the CCI+SCI group (Figure 6D,E, *p* < 0.001). Therefore, we attempted to explore the relationship between PSD‐95 and neuropathic pain.

### 3.7 Increased expression of PSD‐95 in SDH affected phagocytic microglial proliferation

**Figure 7:**
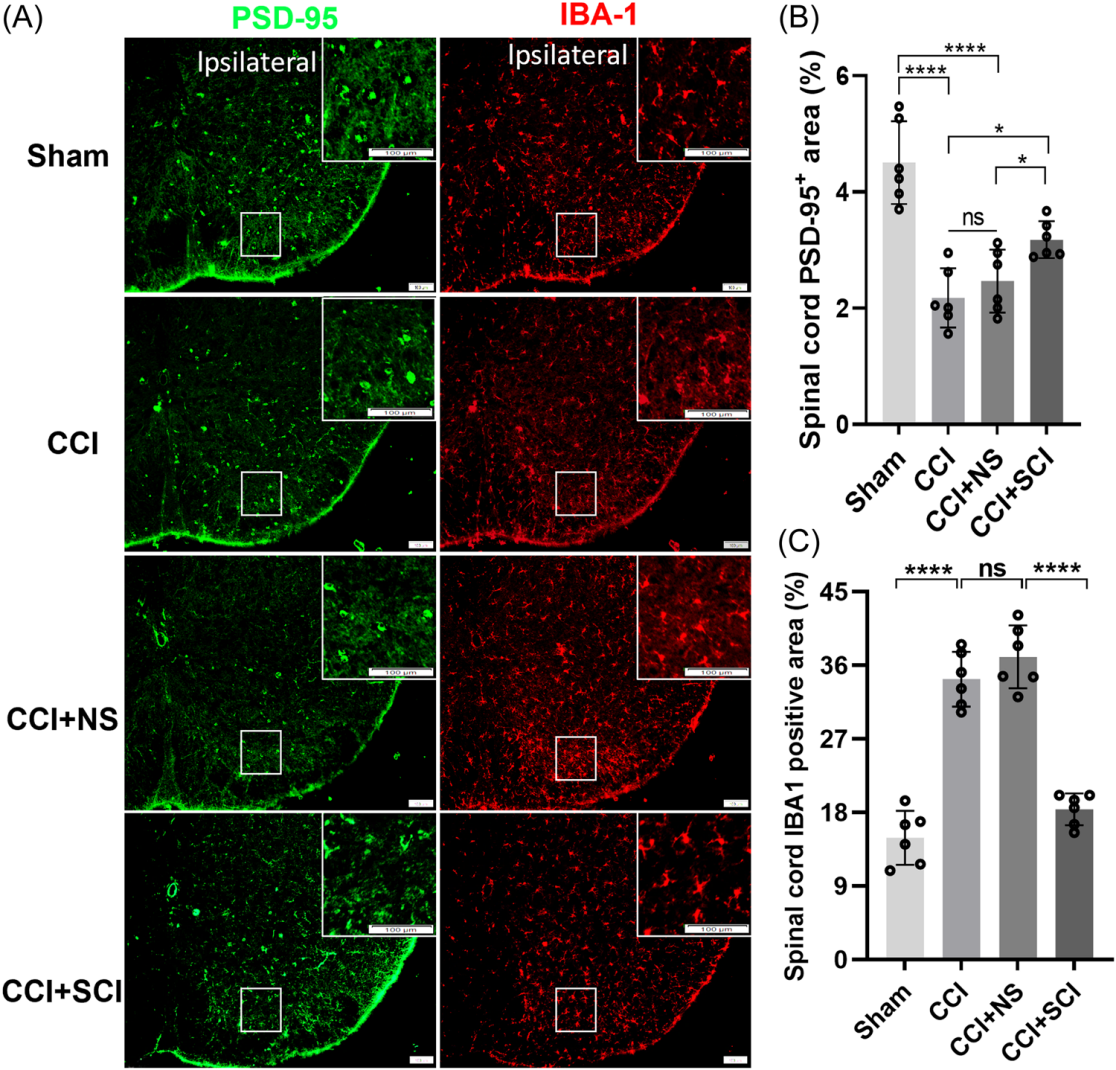
Expression of PSD‐95 and IBA‐1 in the SDH. (A) Colocalization of IBA‐1 (red) and PSD‐95 (green) in the SDH. White rectangles indicate the areas of interest and enlarged images. Scale bars = 100 μm. (B) PSD‐95 expression was quantified in the SDH of the sham, CCI, CCI + PBS, and the CCI + SCI groups. (C) Quantification of colocalization of PSD‐95 with IBA‐1 in the four groups. Scale bars = 100 μm. *n* = 6, mean ± SD, one‐way ANOVA. CCI, chronic constriction injury; IBA‐1, ionized calcium binding adapter molecule 1; ns, not significant; NS, normal saline; PSD‐95, postsynaptic density protein 95; SCI, Stauntonia chinensis injection. *p < 0.05; ****p < 0.0001. [Color figure can be viewed at wileyonlinelibrary.com]

We further observed the expression of PSD‐95 in the SDH of mice in each group and the relationship with microglia by immunofluorescence. Previous studies have shown that PSD‐95 is a postsynaptic scaffolding protein and that phagocytic microglial proliferation can enhance synaptic phagocytosis and reduce PSD‐95 expression. We found that the positive fluorescent area of PSD‐95 was mainly expressed in SDH (Figure 7A), and the expression of PSD‐95 in SDH was significantly decreased after CCI insult compared to the sham group (*p* < 0.0001), which was reversed by SCI treatment in the CCI+NS group (Figure 7A,B, *p* < 0.05). Immunofluorescence results also showed that IBA‐1 was augmented in the SDH of CCI mice (*p* < 0.0001) compared with sham mice, and the proliferation of phagocytic microglial cells induced by CCI was substantially inhibited by SCI treatment (Figure 7A,C, *p* < 0.0001).

## 4 DISCUSSION

In the present study, the results of our study showed that SCI treatment effectively brought about pain relief in CCI mice. Moreover, we discovered that SCI suppressed the activation of microglial cells in the spinal cord of CCI mice, thereby attenuating neuroinflammation. RNA sequencing revealed differential upregulation of PSD‐95 in CCI + SCI mice, and immunofluorescent outcomes demonstrated that local administration of SCI can inhibit the proliferation of phagocytic microglia and increase the expression of PSD‐95 in the SDH. Accordingly, SCI regulated the synaptic phagocytosis of microglial cells by inducing an increase in the expression levels of PSD‐95 in the SDH, resulting in a reduction of spinal neuroinflammation and pain relief in CCI mice.

The clinical manifestations of neuropathic pain caused by peripheral nerve injury in humans are mainly hyperalgesia, paresthesia, and spontaneous pain. CCI mice have neuropathic pain symptoms of hyperalgesia, allodynia, and persistent pain, which has been widely examined in pain research.^25^ In this experiment, we found that CCI induced a significant increase in the frequency of WDR discharges in the spinal cord and a decrease in the pain threshold of CCI mice. In the neuropathic pain model involving spinal nerve ligation, the firing frequency of spinal cord WDR neurons also increased significantly.^26^ However, In CCI + SCI mice, there was no significant change in the firing frequency of WDR neurons in the spinal cord, regardless of whether they were subjected to non‐nociceptive or nociceptive stimuli. SCI inhibited the firing of spinal cord WDR neurons and relieved pain.

In this study, SCI administration adjacent to the injured site of the sciatic nerve was found to inhibit the proliferation of phagocytic microglia. In addition, in vitro experiments showed that LPS‐induced microglial proliferation is an inflammatory proliferation, which was inhibited by SCI in this study. As far as we know, there is hardly any literature documenting the impact of SCI on microglia. After peripheral nerve injury, microglia in the spinal cord were activated, evidenced by increased expression of the microglial markers IBA‐1.^3^ Peripheral nerve injury induced phagocytic microglia in the SDH; microglia showed morphological changes, characterized by retraction and thickening of processes and enlargement of soma.^9^ In this study, CCI‐induced phagocytic microglia show the same morphology. Microglia produce neuromodulators, including cytokines, that rapidly modulate synaptic plasticity. This modulation is a driving force in the pathogenesis of pain after tissue and nerve injury.^27^ In fact, in young animals without a mature immune system, microglial activation is not obvious at the onset of pain until the rat is 4 weeks old,^28^ which explains the pivotal effect of phagocytic microglia in the development of pain in mature animals. Therefore, inhibiting the inflammatory activation of microglia may be one of the methods for treating neuropathic pain, which is one of the mechanisms by which SCI is useful for the treatment of neuropathic pain.

Neuropathic pain is often accompanied by neuroinflammation, and relieving neuroinflammation will effectively reduce pain.^29^, ^30^ Similar to the results of our previous study,^3^ in the present study, we found that CCI mice had significant spinal cord neuroinflammation with increased expression of inflammatory cytokines TNF‐α, IL‐1β, and IL‐6. We used immunofluorescence and found that TNF‐α and IL‐1β colocalized with microglia. In addition, SCI alleviated spinal neuroinflammation in CCI mice and reduced the expression of inflammatory cytokines in the spinal cord. It has been reported that TNF‐α stimulates microglial phagocytosis of beads and live neurons, resulting in neuronal loss in mixed glial–neuronal cultures,^31^ and inhibition of the expression of TNF‐α can significantly relieve pain.^32^ TNF‐α differentially regulates synaptic plasticity in the hippocampus and the spinal cord after peripheral nerve injury by a microglia‐dependent mechanism.^33^ It has been reported that activated microglia release a variety of pro‐inflammatory cytokines during neuropathic pain.^34^, ^35^ Microglia can regulate the transmission of synaptic signals by interacting with a variety of mediators, and then regulate neuroinflammation and control the progression of pain, such as Neuregulin, ERB, matrix metalloproteinases, and chemokine‐mediated pathways.^36^ Moreover, neuroinflammation is characterized by the activation of microglia in the CNS and the release of pro‐inflammatory cytokines that can cause synaptic dysfunction, neuronal death, and neurogenesis inhibition.^37^ In the present study, SCI alleviated neuropathic pain by reducing the proliferation of phagocytic microglia, reducing synaptic phagocytosis, and attenuating neuroinflammation.

Then, by using RNA sequencing, we found that the most significant GO functional enrichment item was modulation of chemical synaptic transmission and the most significant Reactome enrichment pathway was protein–protein interactions at synapses, while PSD‐95 overlapped in these two items. Gene differential analysis also showed that the expression of PSD‐95 was significantly upregulated in CCI+SCI mice. PSD‐95 binds to the NMDA receptor subunit, which can promote downstream intracellular signaling, regulate receptor stability, and promote synaptic plasticity.^38^ Throughout the study, we used Western blot to confirm that the expression of PSD‐95 was increased in CCI + SCI mice. It has been reported that the expression of PSD‐95 in mouse brain is decreased when neuroinflammation is increased.^16^ Similarly, in another study, a reduction in the expression of PSD‐95 is observed in the hippocampus of rats under inflammatory conditions, but an increase in the content of PSD‐95 is found in the hippocampus during the process of ameliorating inflammation.^39^ Pain caused by complete Freunds’ adjuvant injection may lead to increased levels of IL‐6 and decreased levels of PSD‐95 in the mice cortex.^40^ In this study, after SCI treatment, the expression of PSD‐95 in the SDH of CCI mice increased and the proliferation of phagocytic microglia was reduced. Thus, SCI treatment effectively suppressed the proliferation of phagocytic glia and inhibited synaptic phagocytosis by modulating the expression of PSD‐95. Nevertheless, more experiments are needed to understand which components of SCI affect PSD‐95 expression and the physiological mechanisms by which these genes regulate microglia status.

## 5 CONCLUSIONS

In summary, SCI treatment could relieve CCI‐induced neuropathic pain and alleviate neuroinflammation by reducing the proliferation of phagocytic microglia, inhibiting synaptic phagocytosis, and increasing expression of the scaffolding synaptic protein PSD‐95 in the SDH.

## AUTHOR CONTRIBUTIONS

Qian Chen and Song Cao conceived and designed the study; Wenwen Deng, Helin Zou, Senio Campos de Souza, and Li Qian performed the experiments, drafted the manuscript, and analyzed and interpreted the data. Qian Chen and Song Cao finalized the manuscript and reviewed the final version. All authors have read and approved the final manuscript.

## CONFLICT OF INTEREST STATEMENT

Song Cao is an Editorial Board member of Ibrain and a co‐author of this article. To minimize bias, he was excluded from all editorial decision‐making related to the acceptance of this article for publication. The remaining authors declare no conflict of interest.

## ETHICS STATEMENT

The studies involving animals were reviewed and approved by the Laboratory Animal Welfare & Ethics Committee of Zunyi Medical University (ZMU21‐2210‐002).

## Data Availability

Data reported in this study are available from the corresponding author on reasonable request.
